# Comparative evaluation of inductively coupled plasma mass spectrometry and a phosphatase method for lithium measurement: an analytical method-comparison study

**DOI:** 10.3389/fchem.2026.1825328

**Published:** 2026-08-28

**Authors:** Shan He, Jing He, Dan Li, Sihai Ling, Chengeng Liu

**Affiliations:** Beijing Key Laboratory of Intelligent Drug Research and Development for Mental Disorders, National Clinical Research Center for Mental Disorders, National Center for Mental Disorders, Beijing Anding Hospital, Capital Medical University, Beijing, China

**Keywords:** aqueous-standard verification, ICP-MS, lithium measurement, method comparison, phosphatase method

## Abstract

The objective of this study was to compare lithium results obtained by inductively coupled plasma mass spectrometry (ICP-MS) and a phosphatase method in paired patient serum samples and to summarize the available analytical evidence from aqueous-standard verification and routine quality-control records. A total of 171 paired serum samples were included in all method-comparison analyses, comprising 141 non-hemolyzed/non-chylous samples, 17 hemolyzed samples, and 13 chylous samples. ICP-MS analytical response was assessed using traceable aqueous lithium standards, while imprecision was summarized descriptively from routine quality-control records. Matrix-matched trueness verification, spike-recovery, standard-addition, and other serum-specific validation procedures were not performed. Although the two methods showed a strong linear association (r = 0.989), Deming regression demonstrated a constant positive bias of the phosphatase method relative to ICP-MS. At the four predefined concentrations of 0.5, 1.0, 1.2, and 2.0 mmol/L, the predicted phosphatase-method results exceeded the upper bounds of the corresponding acceptable predicted-result intervals. Manufacturer-reported LOD and LOQ values were lower for ICP-MS, but study-specific verification was not performed. These findings identify clinically relevant between-method bias under the analytical conditions of this study but do not constitute full matrix-specific validation of ICP-MS for serum lithium measurement. Prospective matrix-matched validation is required before broader clinical implementation.

## 1 Introduction

Bipolar disorder (BD) is characterized by recurrent manic or hypomanic and depressive episodes and is associated with substantial functional impairment (Nierenberg et al., 2023). The global lifetime prevalence of bipolar-spectrum disorders has been estimated at approximately 2.4% (Merikangas et al., 2011). Major clinical guidelines include lithium among the principal pharmacological treatments for BD (Yatham et al., 2018). Lithium is an established maintenance treatment and reduces the risk of mood-episode relapse (Geddes et al., 2004). Because lithium has a narrow therapeutic range and therapeutic drug monitoring is recommended to guide dose adjustment and reduce the risk of toxicity, reliable serum lithium measurement is clinically important (Grandjean and Aubry, 2009; Hiemke et al., 2018).

Laboratory methods used for serum lithium measurement include ion-selective electrode methods, atomic absorption spectrometry, automated spectrophotometric assays, and ICP-MS. Interlaboratory studies have evaluated ion-selective electrode, reflectance-spectrophotometric, and spectrophotometric platforms and have demonstrated method- and instrument-dependent analytical variation, particularly at lower lithium concentrations (Mose et al., 2015). A spectrophotometric lithium assay has also been implemented on a multichannel chemistry analyzer and compared directly with ion-selective electrode-based measurement for routine testing (Lyon et al., 2004). Because analytical characteristics are method- and platform-dependent, direct comparability should be evaluated using paired patient samples.

ICP-MS provides direct elemental measurement and is used for trace-element quantification in clinical laboratories (Gong et al., 2017; Kojo et al., 2025; Yang et al., 2021). Nevertheless, ICP-MS remains susceptible to matrix suppression or enhancement, instrumental drift, contamination during sample preparation, carryover, inappropriate internal-standard correction, and spectral or non-spectral interferences. Rigorous calibration, internal-standard correction, quality control, and matrix-appropriate verification are therefore required for clinical implementation (Yang et al., 2021). A standard-addition approach has been reported for serum lithium measurement to reduce matrix-related bias (Fan et al., 2022). However, evidence comparing ICP-MS with automated phosphatase-based lithium assays at clinically relevant decision levels remains limited. Accordingly, the present study was designed primarily as a method-comparison study using paired patient serum samples. It also summarizes the available ICP-MS analytical-response verification using aqueous lithium standards and descriptive imprecision estimates derived from routine quality-control records. It was not designed as a complete matrix-specific validation of ICP-MS in human serum, and no spike-recovery, standard-addition, or matrix-matched trueness experiment was performed.

## 2 Subjects and methods

### 2.1 Subjects

A total of 171 patients with mental illness who were treated with lithium carbonate in the Capital Medical University Affiliated Beijing Anding Hospital were included. This study was approved by the ethics committee of the Capital Medical University Affiliated Beijing Anding Hospital. All guardians of the patients provided informed consent and signed a written informed consent form. Inclusion criteria: (1) Patients were diagnosed with mania or BD by a professional physician based on the “Chinese Classification and Diagnostic Criteria of Mental Disorders, Third Edition” (CCMD-3) (Chen, 2002); (2) Patients had normal intelligence and was able to fully understand and cooperate with the physician’s trial requirements; (3) Patients did not exhibit severe self-harm, suicidal ideation, or outwardly aggressive emotions to ensure the safety and smooth progress of the trial. Exclusion criteria: (1) Patients with abnormal blood lipid or blood glucose levels, or accompanied by coagulation dysfunction; (2) Patients with severe infections, central nervous system affective symptoms, or jaundice; (3) During the study, patients who took medications other than lithium carbonate that may affect serum lithium concentration or cause changes.

### 2.2 Sample collection and grouping

Serum samples were collected from 171 patients and categorized according to specimen appearance as 141 non-hemolyzed/non-chylous samples, 17 hemolyzed samples, and 13 chylous samples. All 171 samples were included in the primary paired method-comparison analyses. These specimen categories were recorded descriptively, and no separate category-specific inferential analysis was performed. All samples were aliquoted strictly according to a standard of 0.5 mL, stored at −80 °C, and transported via dry ice cold chain to ensure the quality of the samples was not affected. Based on the detected serum lithium concentration results, the patients were divided into the following four groups. Group A (low-concentration group): lithium concentration < 0.4 mmol/L (n = 38); Group B (therapeutic dose group 1): lithium concentration between 0.4–0.8 mmol/L (n = 61); Group C (therapeutic dose group 2): lithium concentration between 0.8–1.2 mmol/L (n = 38); Group D (high-concentration group): lithium concentration > 1.2 mmol/L (n = 34).

### 2.3 ICP-MS determination

Prior to analysis, frozen serum samples were thawed at room temperature and mixed thoroughly using a vortex mixer. The samples were diluted 20-fold with a diluent containing 0.1% HNO_3_ and 0.01% Triton X-100, followed by centrifugation at 4,500 rpm for 5 min. ICP-MS determination was performed using a Reilipu trace element analyzer (Inspector SQ60) and a lithium determination kit (Reilipu [Hangzhou] Medical Technology Co., Ltd., Hangzhou, China). The Inspector SQ60 is a quadrupole ICP-MS equipped with a kinetic energy discrimination cell and a standard Ni sampler cone. Lithium was quantified by monitoring ^7Li at m/z 7. The proprietary internal-standard solution supplied with the commercial Reilipu lithium determination kit was used in accordance with the manufacturer’s Instructions for Use to correct for instrumental drift and matrix-related signal variation (Reilipu (Hangzhou) Medical Technology Co. L. (2026)). The exact isotope composition, concentration, and addition ratio of the internal-standard solution were not disclosed in the available documentation. External calibration was performed using the lithium calibration materials supplied or specified for the commercial kit, in accordance with the same manufacturer instructions (Reilipu (Hangzhou) Medical Technology Co. L. (2026)).

External calibration was performed according to the manufacturer’s package insert/technical instructions using lithium calibration materials supplied or specified for the commercial ICP-MS kit. The reported ICP-MS calibration range of 0.01–2.00 mmol/L refers to serum-equivalent lithium concentrations after back-calculation for the routine 20-fold dilution. The corresponding diluted measurement-solution range before introduction into the ICP-MS was 0.0005–0.100 mmol/L. The highest recorded ICP-MS result was 2.896 mmol/L, which exceeded the stated serum-equivalent calibration range. No archived documentation was available to confirm whether additional dilution and remeasurement were performed for this sample; therefore, conclusions regarding analytical performance above 2.00 mmol/L were avoided.

ICP-MS operating conditions were as follows: RF generator power, 1,500 W; nebulizer flow rate, 1.04 L/min; auxiliary gas flow rate, 0.82 L/min; cooling gas flow rate, 15.0 L/min; sampling depth, 5.00 mm; and determination mode, KED mode.

The manufacturer-reported/protocol-level ICP-MS LOD and LOQ were 0.002 and 0.006 mmol/L, respectively, expressed as serum-equivalent concentrations after back-calculation for the routine 20-fold dilution. The corresponding diluted measurement-solution equivalents were 0.0001 and 0.0003 mmol/L, respectively. For the phosphatase method, the reported LOD and LOQ were 0.01 and 0.03 mmol/L, respectively, expressed as direct serum concentrations. These values were not independently verified in the present study using replicate blank or low-level serum samples and are therefore presented only as descriptive assay information. They were not used as primary evidence in the comparative performance assessment.

Carryover was monitored as part of routine instrument quality control during calibration and sample analysis. No abnormal blank response requiring corrective action was observed during the analytical runs. However, a prospectively designed quantitative carryover study with a predefined numerical acceptance threshold was not performed.

### 2.4 Phosphatase method

The fully automated biochemical analyzer (model: Beckman AU5800) and the lithium determination kit (Zhongsheng Beikong Biotech Co., Ltd.) were utilized for the detection of serum lithium concentration using the phosphatase method. Prior to the experiment, the biochemical analyzer was calibrated using high, medium, and low concentrations of calibrators provided in the lithium determination kit. Daily internal quality control was performed on both instruments using the Randox quality control products (batch numbers 1104UN and 801UE) to ensure that the results of serum lithium internal quality control were within acceptable limits, thereby guaranteeing the accuracy of all experimental results. Each serum sample was measured twice on both instruments. To reduce possible order effects, the second measurement was performed in the reverse sample sequence. For each method, the arithmetic mean of the two replicate measurements was defined as the final sample-level result. This duplicate-averaged result was used in all subsequent patient-sample method-comparison analyses.

### 2.5 Aqueous-standard verification of ICP-MS analytical response

Verification of the ICP-MS analytical response was performed using traceable aqueous lithium standard solutions at low, medium, and high concentration levels, with the calculation of relative difference adapted from the general framework described in YY/T 1789.2-2021 (Administration NMP, 2021b). These materials were not serum based and were used only to assess the agreement between measured and assigned values under repeatability conditions. The same operator measured each concentration level six times within a short period. Relative difference was calculated as follows: Relative difference (%) = [(mean measured value − assigned value)/assigned value] × 100, where the assigned value was the traceable value of the aqueous lithium standard solution. No serum spike-recovery, standard-addition, matrix-matched trueness, or other serum-specific matrix-effect experiment was performed.

### 2.6 Precision evaluation

Precision was evaluated descriptively using routine lithium quality-control records at low, medium, and high concentration levels (Administration NMP, 2021a). The available records included 30 analytical determinations for ICP-MS and 30 analytical determinations with duplicate readings for the phosphatase method. The available archived summary did not allow reconstruction of individual duplicate pairs or recalculation of CVs using the mean of duplicate readings. Therefore, the phosphatase-method results were reported as “30 analytical determinations with duplicate readings” rather than as 60 independent observations. The coefficient of variation was summarized as a descriptive within-laboratory imprecision estimate. Because the observational units and data structures were not fully standardized between methods, these results were not used for formal statistical comparison of precision superiority. The terminology and coefficient-of-variation calculations were informed by the general precision-evaluation framework described in YY/T 1789.1-2021 (Administration NMP, 2021a). However, the available routine quality-control records did not constitute a prospectively designed precision study fully compliant with that standard.

### 2.7 Evaluation of the consistency ICP-MS and phosphatase methods

The paired patient-sample method comparison between ICP-MS and the phosphatase method was conducted using graphical and regression analyses broadly aligned with the patient-sample comparison framework described in YY/T 1789.2-2021 (Administration NMP, 2021b). Frozen serum samples were thawed at room temperature and mixed thoroughly. Each sample was measured twice by both methods as described above. For each method, the arithmetic mean of the two replicate measurements was used as the paired sample-level observation for the method-comparison analyses. (1) All patient-sample results were reviewed for completeness and consistency with available laboratory records. No result was excluded solely on the basis of high lithium concentration. The sample with ICP-MS = 2.896 mmol/L and phosphatase method = 2.820 mmol/L was retained as a high-concentration patient sample because both methods produced similarly high values and no identifiable analytical error was documented. (2) Graphical analysis: Scatter plots and deviation plots were used to visually demonstrate data distribution, between-method differences, linear ranges, and potential variation trends between the two methods. The scatter plot should display all data, with the x-axis representing ICP-MS test results and the y-axis representing phosphatase test results. Both axes should use the same numerical range and spacing. For the difference plots, the x-axis represents the arithmetic mean of the paired concentrations measured by the two methods. The y-axis represents either the signed difference, calculated as ICP-MS result minus phosphatase-method result, or the corresponding percentage difference.

### 2.8 Statistical analysis

Statistical analysis was performed using MedCalc software (version 20.027). Continuous variables were summarized using descriptive statistics. Pearson correlation analysis was used to describe the linear association between ICP-MS and phosphatase-method results. Pearson correlation was reported only as a descriptive measure of linear association and was not used as evidence of agreement between methods. Pearson correlation, Deming regression, and Bland–Altman analysis were performed using the same 171 paired sample-level results, comprising 141 non-hemolyzed/non-chylous samples, 17 hemolyzed samples, and 13 chylous samples. The observed value entered into the Pearson correlation, Deming regression, and Bland–Altman analyses was the arithmetic mean of the two replicate measurements obtained for each sample on each instrument. The decision-level bias estimates were derived from the Deming regression equation fitted to these 171 paired results. No separate inferential analysis was performed according to specimen category. Formal distributional diagnostics, including histograms, Q-Q plots, residual-versus-fitted plots, residual heteroscedasticity assessment, Spearman correlation, bootstrap confidence intervals, and confidence intervals for Bland–Altman mean bias and limits of agreement, were not prospectively planned. In addition, the row-level paired dataset containing the 171 individual ICP-MS and phosphatase-method results, as well as the original MedCalc project file, could not be recovered from the archived materials available for this revision. The available archived materials included only the exported MedCalc summary output for Pearson correlation and Deming regression, static exported method-comparison figures, summary tables for trueness verification, routine quality-control imprecision, and decision-level bias, and available kit/instrument documentation. Therefore, the additional distributional, residual, nonparametric, and bootstrap analyses could not be recalculated reliably. The correlation, Deming regression, and Bland–Altman findings were interpreted conservatively as descriptive method-comparison results. Because correlation alone does not assess agreement between two analytical methods, method comparison was primarily evaluated using Deming regression, with ICP-MS as the comparator method and the phosphatase method as the test method. Deming regression was performed using MedCalc software, version 20.027, with ICP-MS as the comparator method and the phosphatase method as the test method. The error variance ratio was set to 1 because comparable replicate patient-sample data for estimating method-specific error variances were not available. The routine QC data were not used to derive a formal variance ratio because they were obtained from different materials and non-identical observational structures. Therefore, the Deming regression results were interpreted conservatively. The regression model was expressed as y = bx + a, where x represents the ICP-MS result and y represents the phosphatase-method result. The slope, intercept, and their 95% confidence intervals were reported.

Bias at predefined medical decision levels was estimated from the Deming regression equation. For each ICP-MS decision level, the predicted phosphatase-method result was calculated, and estimated bias was defined as predicted phosphatase-method result minus the corresponding ICP-MS decision level. Deviation plots were used to visually assess the direction and magnitude of between-method differences. All available paired patient-sample results were retained in the primary analysis. The high-concentration patient sample was retained because no identifiable analytical error was documented.

## 3 Results

### 3.1 Aqueous-standard verification results

The aqueous-standard verification results for the ICP-MS system are shown in Table 1. The measured mean values were close to the assigned values at the three tested concentration levels, with relative differences of 0.7%, −0.5%, and 1.5%, respectively. These results describe the analytical response of the ICP-MS system in an aqueous matrix under the tested repeatability conditions. Because the verification materials were aqueous rather than serum based, these findings do not establish trueness, recovery, or matrix-effect performance in human serum. The phosphatase method was not independently evaluated using the same aqueous standard materials.

**TABLE 1:** Verification of the ICP-MS analytical response using traceable aqueous lithium standard solutions.

Repetitions	Standard substance LIA	Standard substance LIB	Standard substance LIC
1	1.85	0.28	2.23
2	1.84	0.28	2.23
3	1.90	0.29	2.24
4	1.87	0.27	2.24
5	1.87	0.28	2.29
6	1.91	0.28	2.28
Average values	1.87	0.28	2.25
References	1.86	0.28	2.22
Deviation values	0.7%	−0.5%	1.5%

### 3.2 Precision evaluation results

As shown in Table 2, the observed CVs from routine quality-control records were numerically lower for ICP-MS than for the phosphatase method, particularly at the low concentration level. However, because the observational units and duplicate-reading structure differed between methods, these data were interpreted as descriptive imprecision estimates rather than as evidence of statistically demonstrated superiority.

**TABLE 2:** Descriptive within-laboratory imprecision of ICP-MS and the phosphatase method based on routine quality-control records.

Descriptive within-laboratory imprecision (CV%)	Low value (no. 694)	Medium value (no. 696)	High value (no. 696)
ICP-MS (n = 30)	2.0%	2.2%	1.4%
Phosphatase method, 30 analytical determinations with duplicate readings	8.3%	2.6%	2.3%

For ICP-MS, the available record contained 30 analytical determinations. For the phosphatase method, the available record contained 30 analytical determinations with duplicate readings. The duplicate readings were not treated as independent observations. Because individual duplicate pairs were not available for recalculating CVs, using duplicate-averaged results, the values are presented only as descriptive routine-QC, imprecision estimates and should not be interpreted as a formal statistical comparison of precision superiority.

### 3.3 Data cleaning and review of serum lithium test results by ICP-MS and phosphatase method

**FIGURE 1:**
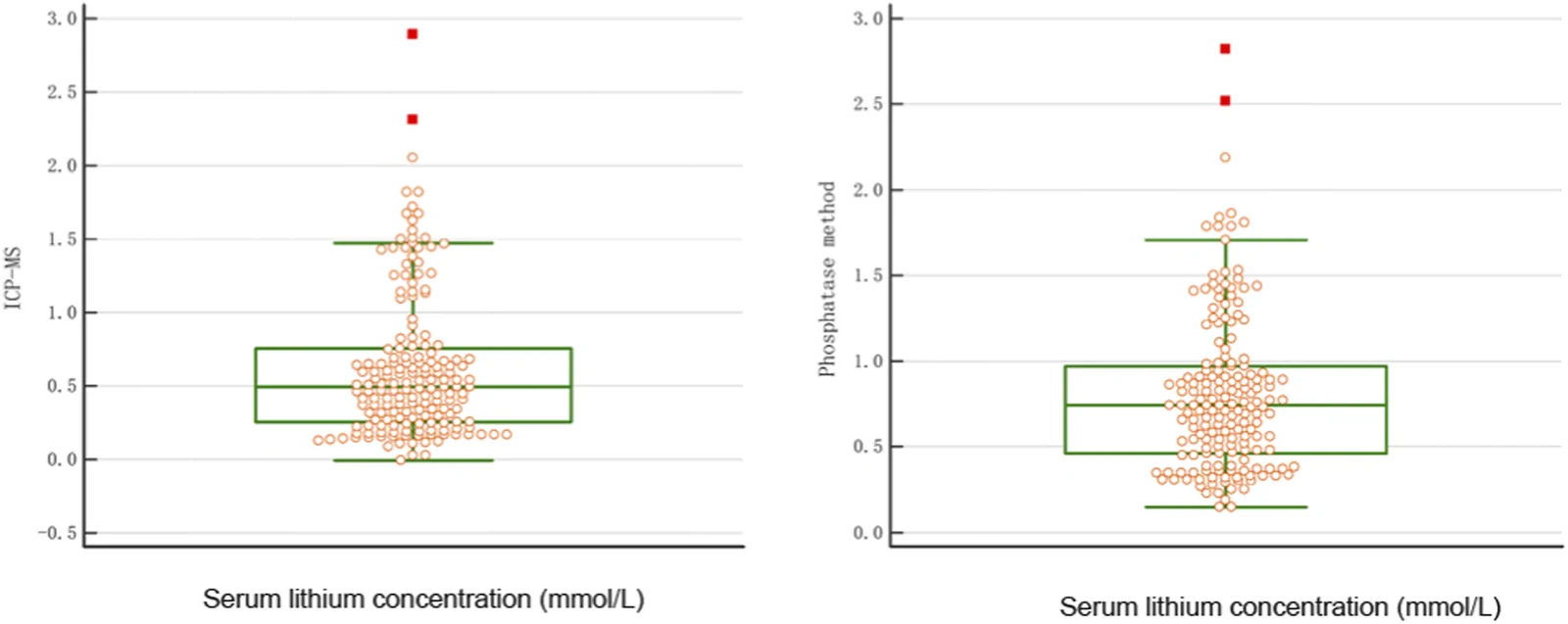
High-concentration serum lithium result measured by ICP-MS and the phosphatase method.

**FIGURE 2:**
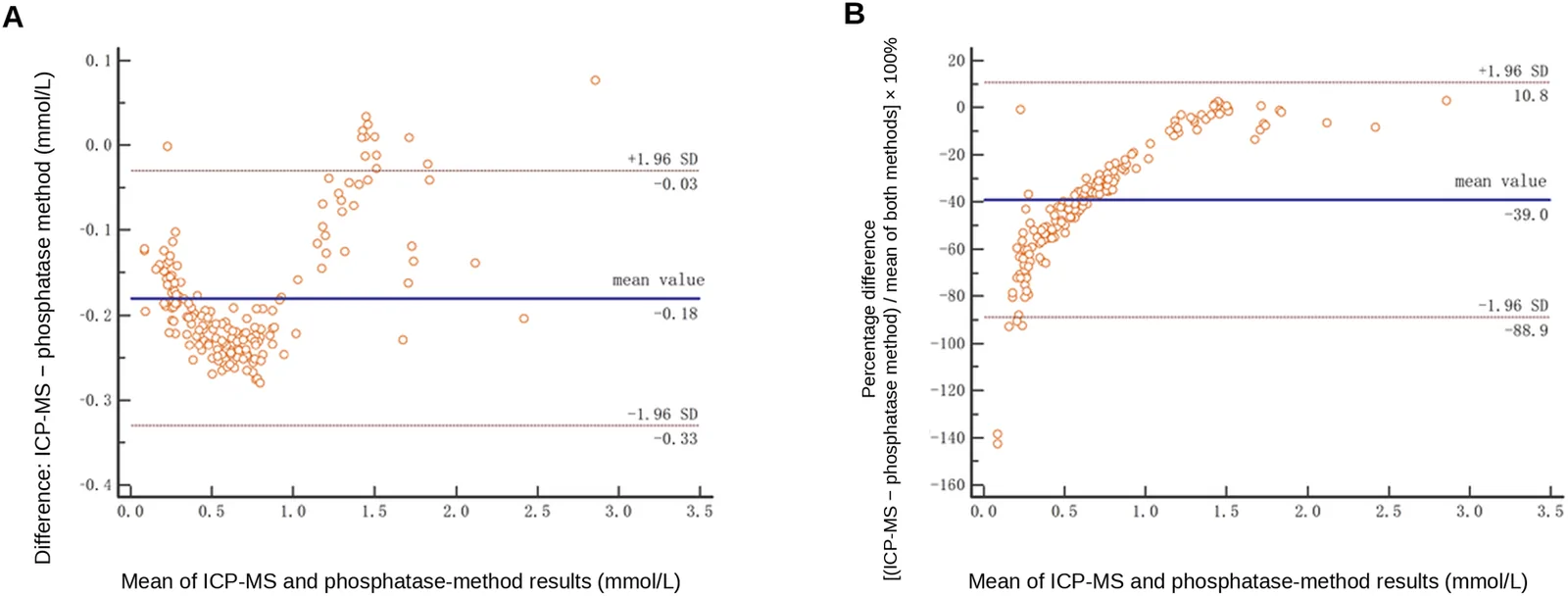
Bland–Altman analysis of 171 paired serum lithium results measured by ICP-MS and the phosphatase method. Each plotted value represents the paired sample-level result used for method comparison. **(A)** Difference plot. The x-axis represents the mean of the paired ICP-MS and phosphatase-method results, and the y-axis represents the signed difference, calculated as the ICP-MS result minus the phosphatase-method result. Therefore, a negative mean difference indicates that the phosphatase method yielded higher results than ICP-MS on average. **(B)** Percentage-difference plot. Percentage difference was calculated as [(ICP-MS result − phosphatase-method result)/mean of the two method results] × 100%. For each method, the plotted sample-level result was the arithmetic mean of two replicate measurements.

Because both methods produced similarly high values, this sample was considered a high-concentration patient sample. Therefore, it was not excluded solely on the basis of its absolute concentration (Figure 1). A very small negative phosphatase result (−0.006 mmol/L) was observed for one low-level sample. This value likely reflected analytical signal fluctuation around the blank or zero-calibrator level after background correction and was below the reported LOQ of the phosphatase method; it was retained as recorded for descriptive analysis, but its clinical interpretation is limited. Potential biological explanations include non-trough blood sampling, recent lithium administration, reduced renal clearance, dehydration, or concomitant medications affecting lithium elimination. Potential analytical causes include sample handling error, dilution error, carryover, or matrix-related interference. No instrument abnormality, empty measurement, or interruption was identified during analysis. Therefore, the result was retained in the primary analysis. Because no formal exclusion-based sensitivity analysis with complete recalculation of Deming regression, Bland–Altman mean bias, limits of agreement, and decision-level bias was available, no sensitivity analysis is reported. Bland–Altman analysis was performed using all 171 paired sample-level results. Figure 2A presents a Bland–Altman difference plot, in which the x-axis represents the mean lithium concentration of each paired measurement and the y-axis represents the signed difference, calculated as ICP-MS result minus phosphatase-method result. Using this definition, the negative mean bias shown in Figure 2A indicates that the phosphatase method yielded higher lithium concentrations than ICP-MS on average. Figure 2B presents the corresponding percentage difference plot, calculated as [(ICP-MS result − phosphatase-method result)/ mean of the two methods] × 100%. The percentage difference plot showed larger relative differences at lower concentrations and smaller relative differences at higher concentrations. This pattern may partly reflect the mathematical effect of a relatively constant absolute bias divided by the mean concentration. Because the 95% CI of the Deming regression slope included 1, statistically clear proportional bias was not confirmed. The main between-method difference was therefore interpreted as constant positive bias. This finding was further evaluated using regression analysis. Because the highest recorded ICP-MS value exceeded the stated serum-equivalent calibration range and no archived documentation confirmed additional dilution and remeasurement, this value was treated as a recorded high-concentration patient result. It was not used to support claims regarding validated analytical performance above 2.00 mmol/L.

### 3.4 Correlation analysis of ICP-MS and phosphatase method

**FIGURE 3:**
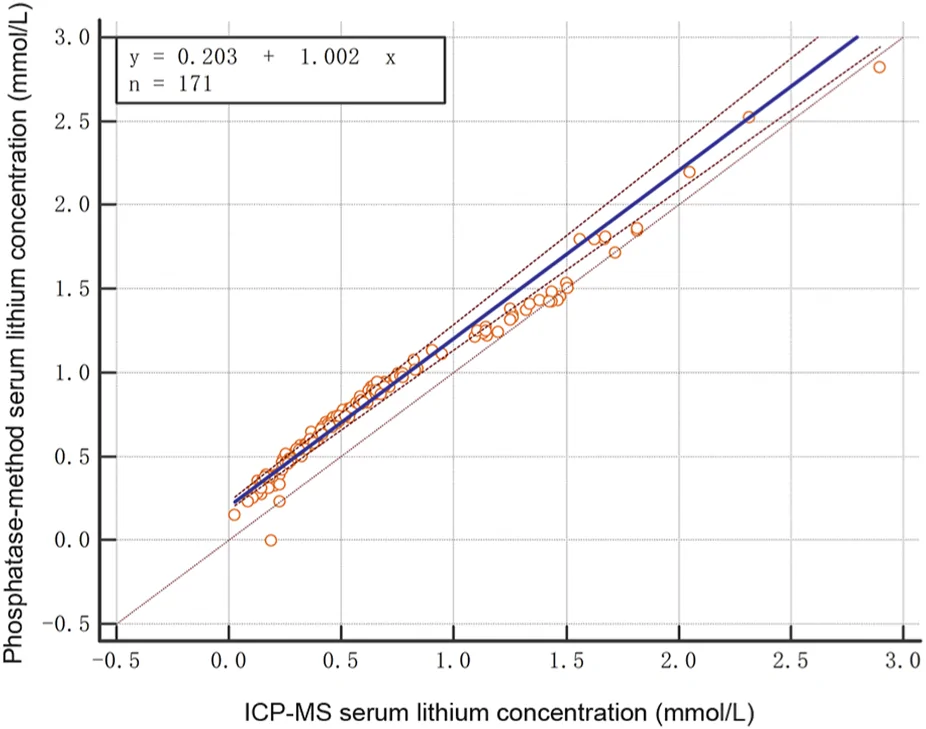
Deming regression analysis of 171 paired serum lithium results measured by ICP-MS and the phosphatase method. ICP-MS was treated as the comparator method and the phosphatase method as the test method. Each point represents one paired sample-level result. For each method, the plotted sample-level result was the arithmetic mean of two replicate measurements.

Deming regression was performed using all 171 paired sample-level results, with ICP-MS treated as the comparator method and the phosphatase method as the test method. The regression equation was y = 0.203 + 1.002x (n = 171; Figure 3). The intercept was 0.203 mmol/L, and its 95% confidence interval did not include zero, indicating a constant positive bias of the phosphatase method relative to ICP-MS. The slope was close to 1, and its 95% confidence interval included 1; therefore, statistically clear proportional bias was not confirmed. Pearson correlation analysis, also based on the same 171 paired results, showed a strong linear association between the methods (r = 0.989; 95% CI, 0.985–0.992). Correlation was interpreted only as a descriptive measure of linear association and not as evidence of agreement or interchangeability.

### 3.5 Assessment of bias at medical decision levels

In this analysis, four predefined lithium concentrations were evaluated: 0.5, 1.0, 1.2, and 2.0 mmol/L. The concentrations of 0.5 and 1.2 mmol/L represent the lower and upper boundaries of the commonly cited therapeutic range. The concentration of 1.0 mmol/L was included as an intermediate concentration within the therapeutic range, and 2.0 mmol/L was included as a high concentration associated with potential toxicity. The acceptable bias criterion was based on one-half of the total allowable error (1/2 TEa; 0.15 mmol/L or 10%, whichever was greater) specified in the 2024 National Center for Clinical Laboratories External Quality Assessment document NCCL-C-01. Based on this allowable-difference criterion, the acceptable predicted-result intervals at predefined ICP-MS concentrations of 0.5, 1.0, 1.2, and 2.0 mmol/L were 0.35–0.65, 0.85–1.15, 1.05–1.35, and 1.80–2.20 mmol/L, respectively. Bias at each predefined ICP-MS concentration was estimated using the Deming regression equation, with ICP-MS treated as the comparator method. The predicted phosphatase-method result was calculated from the regression model, and estimated bias was defined as the predicted phosphatase-method result minus the corresponding ICP-MS concentration. The predicted phosphatase-method results exceeded the upper bounds of the corresponding acceptable predicted-result intervals at all four evaluated concentrations (Table 3). The decision-level estimates were derived from the Deming regression equation fitted to all 171 paired sample-level results and did not represent a separate subset analysis.

**TABLE 3:** Deming regression-based predicted phosphatase-method results and estimated bias at four predefined ICP-MS concentrations.

Predefined ICP-MS concentration (mmol/L)	Predicted phosphatase-method result (mmol/L)	95% CI for predicted result (mmol/L)	Estimated bias (mmol/L)	Acceptable predicted-result interval (mmol/L)	Above acceptable upper limit
0.5	0.70	0.69–0.72	+0.20	0.35–0.65	Yes
1.0	1.21	1.18–1.24	+0.21	0.85–1.15	Yes
1.2	1.41	1.37–1.45	+0.21	1.05–1.35	Yes
2.0	2.21	2.13–2.30	+0.21	1.80–2.20	Yes

Predicted phosphatase-method results were calculated using the Deming regression equation y = 1.002x + 0.203, where x represents the predefined ICP-MS, concentration and y represents the predicted phosphatase-method result. Estimated bias was calculated as the predicted phosphatase-method result minus the predefined ICP-MS, concentration. Acceptable predicted-result intervals were derived using one-half of the total allowable error criterion specified in NCCL-C-01.

## 4 Discussion

This study compared paired patient-serum lithium results obtained using ICP-MS and the phosphatase method and summarized the limited analytical evidence available from aqueous lithium standards and routine quality-control records. It should not be interpreted as a complete validation of ICP-MS for lithium measurement in human serum. The aqueous-standard results assessed analytical response in a non-biological matrix and did not evaluate serum-specific recovery or matrix effects. Similarly, the routine quality-control records provided only descriptive imprecision estimates and were not generated through a prospectively standardized precision-comparison protocol. The principal contribution of the present study is therefore the identification and description of between-method bias in paired patient samples, rather than demonstration of the analytical superiority or interchangeability of either method.

Therapeutic drug monitoring is strongly recommended for lithium because clinical interpretation depends on serum concentration and the conditions under which the sample is collected (Grandjean and Aubry, 2009; Hiemke et al., 2018). In the present study, the available aqueous-standard measurements and routine quality-control records were used only as descriptive supporting information. The aqueous-standard measurements did not assess serum-specific recovery, commutability, or matrix effects. Similarly, the routine quality-control data were not generated using a prospectively standardized protocol with identical observational structures for the two methods. Consequently, these observations do not establish superior accuracy, precision, or reliability of ICP-MS for serum lithium measurement. In this study, the available aqueous-standard measurements and routine quality-control records were used only as descriptive supporting information. The aqueous-standard measurements assessed agreement with assigned values in a non-biological matrix and therefore did not evaluate serum-specific recovery, commutability, or matrix effects. Likewise, the routine quality-control records were not obtained using a prospectively standardized precision protocol with identical observational structures for the two methods. Consequently, these observations do not establish superior accuracy, precision, or reliability of ICP-MS for serum lithium measurement.

The aqueous-standard measurements showed close agreement between measured and assigned values under the evaluated repeatability conditions. However, because the materials were aqueous rather than serum based, these findings describe only the analytical response of the ICP-MS system and do not establish trueness or recovery in human serum. In the descriptive evaluation of routine quality-control records, the observed CVs were numerically lower for ICP-MS than for the phosphatase method, particularly at the low concentration level. Nevertheless, the observational units and duplicate-reading structures differed between methods, and the available data did not support a formal statistical comparison of precision. These findings should therefore be interpreted as descriptive analytical observations rather than evidence that ICP-MS has superior accuracy, precision, reliability, or clinical performance.

In the comparative analysis of 171 paired serum samples, the two methods showed a strong linear association, with a Pearson correlation coefficient of 0.989. However, correlation was interpreted only as a descriptive measure of linear association and not as evidence of agreement or interchangeability. Deming regression yielded a positive intercept of 0.203 mmol/L, indicating a constant positive bias of the phosphatase method relative to ICP-MS. The slope was close to 1 and its 95% confidence interval included 1; therefore, statistically clear proportional bias was not confirmed. At the four predefined concentrations of 0.5, 1.0, 1.2, and 2.0 mmol/L, the predicted phosphatase-method results exceeded the upper bounds of the corresponding acceptable predicted-result intervals shown in Table 3. Accordingly, the two methods should not be considered interchangeable under the analytical conditions evaluated in this study. Potential explanations for the observed bias include differences in calibration traceability, sample matrix effects, and method-specific analytical response. These mechanisms were not directly investigated in the present study and should therefore be regarded as hypotheses requiring prospective evaluation. Because the Bland–Altman difference was defined as ICP-MS result minus phosphatase-method result, the negative mean difference in Figure 2 is directionally consistent with the positive bias of the phosphatase method identified by Deming regression. Nevertheless, because matrix-specific validation of ICP-MS was incomplete, these results do not establish the intrinsic analytical superiority of ICP-MS and should instead be interpreted as evidence of clinically relevant between-method bias requiring prospective confirmation.

Selection of a method for serum lithium measurement should consider the intended clinical use, calibration traceability, quality-control procedures, matrix-specific validation, analytical measurement range, operational complexity, turnaround time, cost, and available laboratory resources. ICP-MS is not free from analytical bias; matrix effects, instrumental drift, sample-preparation contamination, carryover, internal-standard mismatch, and spectral or non-spectral interferences may affect results. ICP-MS implementation therefore requires rigorous calibration, appropriate quality control, validated sample preparation, and matrix-appropriate verification. The phosphatase method offers operational simplicity on automated chemistry platforms, whereas ICP-MS requires more specialized instrumentation and technical control. The present study does not establish that either method is intrinsically superior.

Several limitations should be acknowledged. First, a certified serum reference material with an assigned lithium concentration was not available; therefore, trueness verification was performed using traceable aqueous lithium standard solutions rather than matrix-matched serum reference materials. Accordingly, the present study should be regarded as a patient-sample method-comparison study with limited aqueous-standard verification rather than as a complete matrix-specific validation of ICP-MS for serum lithium measurement. In addition, the highest recorded ICP-MS result exceeded the stated serum-equivalent calibration range. Because no archived documentation confirmed additional dilution and remeasurement for this sample, analytical performance above 2.00 mmol/L could not be verified in this study. Second, the phosphatase method was not independently verified for trueness using the same standard materials and was evaluated mainly as the comparator method in patient-sample comparison. Third, the reported LOD and LOQ values were based on validated assay protocols and were not independently recalculated using replicate blank and low-level serum samples in this study. Fourth, quantitative carryover testing with a predefined numerical threshold and systematic ruggedness assessment were not included in the original protocol. Fifth, the internal standard was used according to the commercial kit instructions, but the exact isotope composition was not available in the laboratory record. Another important limitation is that the de-identified row-level paired dataset and the original MedCalc project file were not available for the present revision. Only exported statistical summaries, static figures, summary tables, and available laboratory documentation were retained in the archived materials. Therefore, additional analyses requiring individual paired observations, including histograms, Q-Q plots, residual-versus-fitted plots, residual heteroscedasticity assessment, Spearman correlation, bootstrap confidence intervals, and confidence intervals for Bland–Altman mean bias and limits of agreement, could not be recalculated. Finally, this was a single-center analytical study and did not evaluate clinical outcomes, cost-effectiveness, turnaround time, or inter-laboratory reproducibility. Details required for full reproducibility of the ICP-MS method were incomplete in the archived records. Although the manufacturer’s package insert/technical instructions for the Reilipu lithium determination kit were reviewed, the exact composition, isotope, concentration, and addition ratio of the proprietary internal-standard solution were not disclosed in the available documentation. In addition, the catalog number, lot number, package-insert version/date, number of calibration points, exact concentration of each calibration point, calibration matrix, blank type, regression model, weighting, calibration acceptance criteria, and complete traceability chain could not be verified from the archived laboratory records. These limitations constrain the reproducibility of the ICP-MS method as reported in this study. Confidence intervals for the Bland–Altman mean bias and limits of agreement could not be recalculated because the row-level paired dataset and the original statistical project file were not available. Only the exported static Bland–Altman figures and summary analysis outputs were retained in the archived materials.

## 5 Conclusion

In this single-center analytical method-comparison study, ICP-MS and the phosphatase method showed a strong linear association, but the phosphatase method demonstrated a constant positive bias relative to ICP-MS at the evaluated concentrations. The aqueous-standard and routine quality-control findings provide limited descriptive analytical evidence only and do not constitute matrix-specific validation of ICP-MS in human serum. Accordingly, the two methods should not be considered interchangeable under the evaluated analytical conditions, and the present data do not support a definitive claim of analytical superiority for either method. Prospective matrix-matched validation, including recovery and matrix-effect assessment, quantitative carryover testing, standardized precision studies, and multicenter evaluation, is required before broader clinical implementation.

## Data Availability

The original contributions presented in the study are included in the article/supplementary material, further inquiries can be directed to the corresponding author.
